# Oral Presentation - Abstracts of the 25^th^ Annual Congress of the SBRA and the 15^th^ REDLARA Taller General Rio de Janeiro/RJ - Brazil, 2021

**DOI:** 10.5935/1518-0557.20210106

**Published:** 2022

**O-01. Pharmacological tools to induce endometrial regeneration through endogenous mobilization of mesenchymal stem cells**

**Serena Rossato** ^**1**^ **, Rafaela Dib Ishimura** ^**1**^ **, Julio Cesar Rosa e Silva** ^**1**^ **, Ana Carolina Japur de Sá Rosa e Silva** ^**1**^

^*1*^ *Centro de Reprodução Humana do Hospital das Clínicas de Ribeirão Preto - USP.*

**Objective:** Thin endometrium (TE) represents a huge challenge in assisted reproduction, since it is related to decreased endometrial receptivity and consequent implantation failure, negatively affecting reproductive outcomes. The application of mesenchymal stem cells (MSE) has shown promising results, as its regenerative properties improves thickness and expression of receptivity biomarkers in the endometrium. However, the costs and complex processes involved in these treatments prevent its routine aplication. In this context, there is a need to create strategies to stimulate specific stem cell recruitment and migration to tissue injury sites. AMD3100 is a CXCR4 antagonist receptor that disrupts the CXCL12-CXCR4 axis - which is directly related to stem cell migration - and has been used in tissue regeneration studies to increase the influx of stem cells to the site of injury. In addition, its combined use with β3 adrenergic agonists receptors leads to selective recruitment of MSC with better regenerative results proved in a recent paper on bone fracture healing. Thus, the objective of this study is to evaluate the regenerative potential of AMD3100 alone or in association with a β3 adrenergic agonist receptor in a murine animal model of TE and its impact on the reproductive outcomes.

**Methods:** Experiments were carried out on female C57Bl/6 mice (age 9 weeks; 20 g). TE was induced by direct application of 50 µl of 95% ethanol in the exposed uterus through an abdominal incision, while Phosphate-buffered saline(PBS) was used in the sham procedure (SH). Mice in each group were randomly divided into 3 treatment subgroups (n = 8 for each): (1)PBS(PL); (2)AMD 3100(5 mg/kg sc) single dose after surgery (AMD); (3)BRL37344(rodent-selective β3 adrenergic receptor agonist)(10 mg/kg sc) for 4 consecutive days previous to surgery plus AMD 3100(5 mg/kg sc) single dose (AMD/B3). Two estrous cycles after modeling and treatment, all the groups were mated with proven male C57BL/6 mice. The pregnancy rate and litter size were evaluated in order to establish the impact on the reproductive outcomes. In addition, a macroscopic anatomic analysis was performed on the offspring to evaluate possible teratogenic effects.

**Results:** In relation to pregnancy rate, 23 out of 24 sham mice got pregnant (despite the treatment group). The TE model reproduced the literature results, with under 30% of failure in inducing endometrial damage, represented by a pregnancy rate of 25%(2/8) in the TE+PL group in comparison to all sham groups(*p*<0.05). The AMD/B3 treatment completely restored the endometrial function, promoting pregnancy rates similar to all sham groups(*p*>0.05). There was no difference between the TE+AMD treatment group and all the other groups(*p*>0.05) in terms of pregnancy, although a clinically relevant difference between TE+PL and TE+AMD groups was found(25% versus 62.5%, respectively). The litter size was similar between the treatment groups, except for the TE+AMD group with a smaller number of newborns. Regarding the teratogenic potential of the tested drugs (AMD and B3), we did not observe any relevant macroscopic malformations (internal and external) in the treated groups.

**Conclusion:** Our findings indicate a potential therapeutic option for infertility related to thin endometrium. The combination treatment with AMD3100 and β3 adrenergic agonist receptor resulted in an expressive improvement in reproductive outcomes, without a detrimental impact on the offspring development and anatomy. Moreover, this treatment utilises two clinical approved classes of drugs, which could reduce the timeline and cost of translational therapeutic applications in the future.

**O-02. Maternal and neonatal outcomes after frozen, fresh and ovulation induction cycles: are there any differences?**

**Mariana Barth**^**1**^ **, Marta Ribeiro Hentschke**^**2**^**, Victoria Campos Dornelles**^**2**^**, Isadora Badalotti-Teloken**^**2**^**, Talita Colombo**^**2**^**, Alvaro Petracco**^**2**^**, Bartira Pinheiro da Costa**^**1**^**, Mariangela Badalotti**^**2**^

^*1*^ *Fertilitat - Centro de Medicina Reprodutiva.*

^*2*^ *Pontifícia Universidade Católica do Rio Grande do Sul.*

**Objective:** To evaluate maternal and neonatal outcomes in pregnancies resulted from frozen embryo transfers (FET), fresh embryo transfers (IVF-ET), and ovulation induction (OI) cycles.

**Methods:** Retrospective cohort study performed at an assisted reproductive clinic in southern Brazil, with data collected from electronic records from 2013 to 2020. A total of 933 single clinical pregnancies were included. Sample was divided into Group 1, FET (n= 123), Group 2, IVF-ET (n= 658), and Group 3, OI (n= 152). maternal and neonatal variables were compared between groups and were expressed as mean±SD or n(%). ANOVA, Tukey test and X^2^ were applied. Data were adjusted for maternal age. Statistical significance was defined as *p*<0.05.

**Results**: When comparing G1 vs. G2 vs. G3, the following results were found, respectively: maternal age (35.7±4.5 *vs*. 35.2±3.7 *vs*. 33.9±3.8, *p*<0.001); pre-eclampsia (3.4% *vs*. 3.4% *vs*. 0.8%, *p*=0.307); placenta disorders (2.2% *vs*. 0.8% *vs*. 0.8%, *p*=0.439); premature rupture of membranes (0% *vs*. 1.8% *vs*. 2.5%, *p*=0.367); gestational diabetes (0% *vs*. 0.4% *vs*. 0%, *p*=0.653); HELLP syndrome (0% *vs*. 0.2% vs 0%, *p*=0.809); live birth (per clinical pregnancy) (72.4% *vs*. 75.5% *vs*. 80.3%, *p*=0.290). According to neonatal outcomes, the following results were found: low birth weight (5.7% *vs.* 10.9% *vs*. 7.3%, *p*=0.312); macrosomic newborns (5.7% vs. 2.0% *vs*. 3.3%, *p*=0.312); Apgar score >7 in fifth minute (100% *vs*. 98.4% *vs*. 99.2%, *p*=0.406), prematurity rate (6.8% *vs*. 9.5% *vs*. 4.1%, *p*=0.134); extreme prematurity rate (< 34 weeks) (1.1% *vs*. 5.1% *vs*. 5.7%, *p*=0.134); small for gestational age (SGA) newborns (4.3% vs. 15.3% vs. 7.5%, *p*=0.011); appropriate for gestational age (AGA) newborns (89.9% *vs*. 80.1% *vs*. 89.7%, *p*=0.022); large for gestational age (LGA) newborns (5.8% vs. 4.6% vs. 2.8%, *p*=0.608); congenital malformations (0% *vs*. 1.4% *vs*. 1.6%, *p*=0.509 ), postnatal death (0% *vs*. 2.0% *vs*. 0%, *p*=0.117), and neonatal intensive care unit rate (3.4% *vs*. 8.4% *vs*. 7.4%, *p*=0.253).

**Conclusion:** In this study, SGA babies were significantly higher after IVF-ET. This finding highlights the importance of paying attention to the birth percentile after FET and IVF-ET pregnancies, with IVF-ET pregnancies being related to the highest percentage of SGA babies and the OI group being related to the highest percentage of AGA babies. Even with no impact on other immediate neonatal outcomes after these infertility treatments, these should be considered. Data on maternal outcomes and their correlation with neonatal outcomes did not show differences in this study, but more studies should be performed for better conclusions.

**O-03. Prediction of fetal heartbeat through artificial intelligence and morphological, morphokinetics and patient variables**

**Dóris Spinosa Chéles** ^**1,2**^ **, Eleonora Inácio Fernandez** ^**2**^ **, André Satoshi Ferreira** ^**2**^ **, Lorena Bori3, Marcos Meseguer** ^**3,4**^ **, José Celso Rocha** ^**5**^ **, Marcelo Fábio Gouveia Nogueira** ^**1,5**^

^*1*^ *São Paulo State University (UNESP), Institute of Biosciences, Department of Pharmacology, Botucatu, Brazil.*

^*2*^ *São Paulo State University (UNESP), School of Sciences and Languages, Assis, Brazil.*

^*3*^ *IVF laboratory, IVI Valencia, Valencia, Spain.*

^*4*^ *Health Research Institute la Fe, Valencia, Spain.*

^*5*^ *São Paulo State University (UNESP), Department of Biological Sciences, School of Sciences and Languages, Assis, Brazil.*

**Objective:** Evaluate the feasibility of variables originated from blastocyst morphology, embryonic development morphokinetics and variables related to assisted reproduction patients being predictive of pregnancy, when used as inputs for a software based on artificial intelligence (AI).

**Methods:** The database used was provided by the IVI Valencia clinic, Spain. Images were obtained through time-lapse system (EmbryoScope^®^ equipment). Digital image processing was performed to automatically extract 33 predictive variables of blastocyst’s morphology. In addition, 16 variables related to embryonic morphokinetic and 4 variables related to patient characteristics were used. These variables were applied separately and together as inputs for a software based on AI (artificial neural networks, ANNs) combined with genetic algorithm for pregnancy (detection of fetal heartbeat) prediction. All these procedures were performed in the MatLab^®^ platform.

**Results:** ANNs were trained for of the three variable input sets (single or together; morphology, morphokinetics and patient). The best ANNs found were those related to morphology, morphokinetics, and the three sets together. For the morphology (33 variables as inputs to ANNs), morphokinetics (16 variables as inputs) and the morphology plus morphokinetics and patient (53 variables) sets, the best training accuracies were, respectively, 94, 94 and 91% for positive pregnancy prediction and, respectively, 90, 97 and 98% for negative pregnancy prediction.

**Conclusion:** Even with distinct sources of the used variables, accuracies above 90% demonstrated the capacity of ANNs in predicting gestational success. These results demonstrate the potential of AI as an objective and reproducible tool to assist embryologists in selecting the fittest embryo. It could, in due course, increase the pregnancy rate currently obtained in assisted reproductive clinics.

**O-04. Time-lapse monitoring: an adjunct tool to select embryos for preimplantation genetic testing**

**Daniela Paes de Almeida Ferreira Braga** ^**1,2**^**, Amanda Souza Setti**^**1,2**^**, Livia Silva Vingris**^**1,2**^**, Patricia Guilherme**^**1,2**^**, Assumpto Iaconelli Jr.**^**1,2**^**, Edson Borges Jr.**^**1,2**^

^*1*^ *Chromosome Medicina Genômica Laboratory, São Paulo, SP, Brazil.*

^*2*^ *Chromosome Medicina Genômica Laboratory, São Paulo, SP, Brazil.*

**Objective:** Over the last decades, efforts have been made to assess the genetic status of preimplantation embryos, distinguishing chromosomally normal embryos from their aneuploid siblings. However, the invasive nature of the technique limits its success. Given the challenges associated with invasive embryo biopsy, ongoing studies are now seeking for non-invasive assessments of the embryo DNA. The identification of morphokinetics events affected by embryo ploidy may be a powerful toll in assisted reproduction, improving the selection of the embryo with the highest potential for implantation, without the detrimental effects of embryo biopsy. Thus, the aim of this study was to investigate the effect of embryo aneuploidy on embryo morphokinetics events in a time lapse imaging (TLI) culture system.

**Methods:** This case-control study was performed in a private university-affiliated IVF center, between 2019 March and December 2020. Kinetic data were analyzed in 957 embryos, derived from 316 patients undergoing preimplantation genetic testing for aneuploidy PGT-A, individually cultured in a TLI incubator until day five of development. Timing of morphokinetic variables, the incidences of multinucleation, and known implantation data score (KIDScore) day 5 were compared between euploid (n=352), aneuploid embryos (n=583), and mosaic embryos (n=22).

**Results:** Aneuploid embryos showed significantly longer tPNf, t2, t3, t4, t6, t7, t8, tM, tsB, and tB compared to euploidy embryos. Although the time to complete t2-tPNf synchronous divisions (s1) were equal among the groups, aneuploid embryos showed significantly longer timing to complete t4-t3 synchronous divisions (s2) and t8-t5 synchronous divisions (s3). The KIDScore day 5 also ranked significantly different between euploid and aneuploid embryos, where euploidy embryos showed a significantly higher score when compared to the aneuploidy ones. No significant differences were noted in the incidences of multinucleation at 2-cell or 4-cell stages. Mosaic embryos behaved similarly to aneuploid embryos in terms of morphokinetic events. The regression analysis showed an increased chance of being mosaic among embryos with longer tPNf, t2, t3, t6, t7, tM, tB. The same results were observed for KIDScore day 5. In a further analysis, embryos were hierarchically distributed into four quartiles according with the KIDScores D5: Q1 with KIDScore D5 ≤3.9, Q2 with KIDScore D5 between 4 and 5.6, Q3 with KIDScore D5 between 5.7 and 7.5, and Q4 with KIDScore ≥7.6. The incidence of euploid embryos were significantly different among the KIDScore D5 category groups, in which the percentage of euploid embryos gradually increased as the KIDScore D5 increased. Finally, the cycles were split into two groups according with the maternal age. For patients > 35 years old, the logistic regression analysis demonstrated that, comparing with embryos in the Q4, embryos in the Q1, Q2, and Q3 have a decreased chance of being euploid (20.2%, 33.7%, 35.5%, and 48.7%, for Q1, Q2, Q3 and Q4 respectively, *p*<0.001). For patients≤ 35 years old, comparing with embryos in the Q4, embryos with KIDScore D5 in the Q1 and Q2, but not in Q3, have a decreased chance of being euploid (35.7%, 44.4%, 61.5%, and 65.2%, for Q1, Q2, Q3 and Q4 respectively, *p*<0.001).

**Conclusion:** A significant effect of aneuploidy on early and late embryos morphokinetic events was observed. Apparently, mosaic embryos behave similarly to aneuploid embryos in terms of morphokinetics, but further studies with a larger number of embryos will be able to confirm these findings more reliably. TLI monitoring may be an adjunct approach to select embryos for PGT.

**O-05. Prediction of Seminal Parameters Improvement after Varicocelectomy using 1H NMR-based Metabonomics Assays**

**Filipe Tenorio Lira Neto** ^**1,2,3**^ **, Ronmilson Alves Marques** ^**4**^ **, Alexandre de Freitas Cavalcanti Filho** ^**2**^ **, João Eduardo Freire da Fonte** ^**5**^ **, Salvador Vilar Correia Lima** ^**3**^ **, Ricardo Oliveira Silva** ^**4**^

^*1*^ *Andros Recife, Recife, Brazil.*

^*2*^ *Department of Urology, Instituto de Medicina Integral Prof. Fernando Figueira, Recife, Brazil.*

^*3*^ *Departament of Surgery, Universidade Federal de Pernambuco. Recife, Brazil.*

^*4*^ *Department of Fundamental Chemistry, Universidade Federal de Pernambuco. Recife, Brazil.*

^*5*^ *Department of Radiology, Real Hospital de Beneficiência Portuguesa, Recife, Brazil.*

**Objective:** To create metabolomics models capable of segregating men who will improve semen analysis (SA) parameters after microsurgical varicocelectomy (MV) from those who will not, using Hydrogen-1 nuclear magnetic resonance (1H NMR) spectra of seminal plasma of pre-operative samples.

**Methods:** We recruited 29 infertile men with palpable varicocele scheduled for MV. 1H NMR spectra of seminal plasma were obtained from pre-operative samples and used to create metabonomics models to discriminate between men who improved and did not improved SA parameters after MV. Improvement was defined as an increase in the total motile progressive sperm count (TMC) of the post-operative SA when compared to the baseline.

**Results:** Most baseline parameters showed no difference between the groups; however, I group participants had a longer duration of infertility, and lower LH levels. The patients of I group showed significant improvements in median sperm concentration (3.0±11.1x10^6^/mL *vs*. 16.0±19.0x10^6^/mL, *P*-value <0.05), median total sperm count (8.5±19.7x10^6^ *vs*. 41.0±54.0x10^6^, *P*-value <0.05), mean progressive motility (26%±15% *vs*. 36%±15%, *P*-value <0.05), and median TMC (1.7±7x10^6^ *vs*. 14.7±28.9x10^6^, *P*-value <.05). Conversely, the NI group had a significant decrease in the median TMC (1.9±23.0 x10^6^ *vs.* 1.1±11.4x10^6^, *P*-value <0.05), and non-significant decreases in median sperm concentration (5.0±11.0 x 106/mL vs 1.0±6.0 x106/mL, *P*-value=0.31) and median total sperm count (12.5±36.0x10^6^ *vs.* 5.0±30.0 x10^6^, *P*-value=0.06) When the postoperative SA parameters were compared between the two groups, the I group showed higher median sperm concentration, median total sperm count and median TMC than the NI group as expected. Regarding the changes in the TMC from baseline, the I group had a median increase of 12.2±23.8x10^6^, whereas the NI group had median decrease of -1.7±9.1x10^6^ (*P*-value <0.05). When the changes were analyzed using percentage, this difference was further highlighted (I group median increase of 275±13% vs. NI group median decrease of -59±7%; *P*-value <0.05). Using Linear Discriminant Analysis, we created a model that discriminated the two groups with sensibility, specificity and accuracy values equals to 94.4%, 90.9% and 93.1% respectively. We identified 4 metabolites that were important for group segregation: caprylate, isoleucine, glycerol-3-phosphocoline, and n-acetyltyrosine.

**Conclusion:** We demonstrated that participants whose SA parameters improved after the procedure displayed different preoperative seminal metabolome profile from those who did not improve. In addition, we created metabonomics model to predict the result of MV in infertile men with varicocele with an accuracy of 93%. Furthermore, the most important metabolites for group segregation are involved in energy metabolism and oxidative stress response, reinforcing the pivotal role of these mechanisms in the pathophysiology of varicocele1H NMR spectroscopy of seminal plasma can be used in conjunction with multivariate statistical tools to create metabonomics models useful in infertile men with varicocele to discriminate those who will improve SA parameters after varicocele from those who will not. This finding has clinical application, helping in the counseling of infertile men with varicocele regarding their prognosis after surgery.

